# Molecular Epidemiology and Genetic Diversity of the Enteric Protozoan Parasite *Blastocystis* sp. in the Northern Egypt Population

**DOI:** 10.3390/pathogens12111359

**Published:** 2023-11-15

**Authors:** Doaa Naguib, Nausicaa Gantois, Jeremy Desramaut, Nagah Arafat, Mohamed Mandour, Asmaa Kamal Kamal Abdelmaogood, Ashraf Fawzy Mosa, Constance Denoyelle, Gaël Even, Gabriela Certad, Magali Chabé, Eric Viscogliosi

**Affiliations:** 1 CNRS, Inserm, CHU Lille, Institut Pasteur de Lille, U1019–UMR 9017–CIIL–Centre d’Infection et d’Immunité de Lille, University of Lille, F-59000 Lille, France; doaanaguib246@yahoo.com (D.N.); nausicaa.gantois@pasteur-lille.fr (N.G.); jeremy.desramaut@pasteur-lille.fr (J.D.); constancedenoyelle@gmail.com (C.D.); gabriela.certad@pasteur-lille.fr (G.C.); magali.chabe@univ-lille.fr (M.C.); 2 Department of Hygiene and Zoonoses, Faculty of Veterinary Medicine, Mansoura University, Mansoura 35516, Egypt; 3 Department of Poultry Diseases, Faculty of Veterinary Medicine, Mansoura University, Mansoura 35516, Egypt; nagaharafat@yahoo.com; 4 Clinical Pathology Department, Faculty of Medicine, Suez Canal University, Ismailia 41511, Egypt; mohamed_mostafa@med.suez.edu.eg (M.M.); asmaa.kamal@med.suez.edu.eg (A.K.K.A.); 5 Parasitology Department, Medical Research Institute, Alexandria University, Alexandria 21500, Egypt; ashraf_fawzy95@yahoo.com; 6 GD Biotech-Gènes Diffusion, F-59000 Lille, France; g.even@genesdiffusion.com; 7 PEGASE-Biosciences (Plateforme d’Expertises Génomiques Appliquées aux Sciences Expérimentales), Institut Pasteur de Lille, F-59000 Lille, France; 8 Délégation à la Recherche Clinique et à l’Innovation, Groupement des Hôpitaux de l’Institut Catholique de Lille, F-59000 Lille, France

**Keywords:** *Blastocystis* sp., Egypt, humans, molecular epidemiology, transmission sources, zoonosis

## Abstract

*Blastocystis* sp. is currently reported as the most frequent single-celled eukaryote inhabiting the intestinal tract of humans and a wide range of animal groups. Its prevalence is especially higher in developing countries linked with fecal peril. Despite a growing interest in this enteric protozoan, certain geographical regions potentially at high risk of infection, such as North Africa, remain under-investigated. Therefore, a large-scale molecular epidemiological survey, including 825 participants presenting digestive disorders or not, was conducted in five governorates located in Northern Egypt. A real-time polymerase chain reaction (qPCR) assay was performed to identify the parasite in stool samples, followed by direct sequencing of the positive PCR products for subtyping and genotyping of the corresponding isolates. The overall prevalence was shown to reach 72.4% in the Egyptian cohort, coupled with a variable frequency depending on the governorate (41.3 to 100%). Among the 597 positive participants, a large proportion of them (39.4%) presented mixed infections, as determined by sequencing. The remaining individuals with single infection were predominantly colonized by subtype 3 (ST3) (48.3%) followed by ST1 (39.5%), ST2 (10.8%), ST14 (1.1%), and ST10 (0.3%). This was the first report of ST10 and ST14 in North Africa. Age, sex, digestive symptoms, and health status of the participants or contact with animals were not identified as significant risk factors for *Blastocystis* sp. occurrence or affecting the ST distribution. In contrast, substantial variations in the prevalence and ST distribution of the parasite were reported according to the governorate. Genotyping of isolates revealed the lower intra-ST diversity for ST3, followed by ST1 and then ST2. By combining subtyping and genotyping data, a widespread inter-human transmission was strongly suggested for ST3 within the Egyptian cohort. Regarding ST1 and ST2, additional animal or environmental sources of infection by these STs have been proposed, whereas the few cases of colonization by ST10 and ST14 were likely the result of zoonotic transmission from bovid. These investigations clearly emphasized the active circulation of *Blastocystis* sp. in Northern Egypt and the necessity for health authorities to implement prevention campaigns towards the population and quality control of drinking water, with the aim of reducing the burden of this enteric protozoan in this endemic country.

## 1. Introduction

*Blastocystis* sp. is currently the most frequent unicellular eukaryote inhabiting the gastrointestinal tract of humans, as reported in numerous epidemiological surveys [1,2,3,4]. Therefore, its prevalence may exceed 50% in many developing countries, particularly on the African continent [5,6], and the number of individuals affected by this parasite is expected to reach 1 billion worldwide [7]. The high frequency of *Blastocystis* sp. in a particular geographical area is directly correlated to the encountered insufficient sanitary and hygienic conditions. Therefore, the main mode of transmission of the parasite is the fecal–oral route, primarily by direct human-to-human contact and through the consumption of water contaminated with environmentally resistant cystic forms excreted by human and various animal hosts [8]. Indeed, *Blastocyctis* sp. is capable of colonizing a broad range of animals, from mammals to insects, fish, and reptiles [2,9,10,11,12], some of which serve as significant reservoirs of zoonotic transmission, as previously evidenced for pigs [13], chicken [14], and non-human primates [15].

Within the genus *Blastocystis*, extensive genetic diversity has been uncovered between morphologically similar isolates, as reported from comparative genomic data [16]. Based primarily on polymorphisms within the small subunit ribosomal RNA (SSU rRNA) gene, 44 lineages referred to as subtypes (STs) (possibly separate species) have been proposed so far [17,18,19,20,21]. However, four of them are not yet validated because they may be due to experimental artifacts [22]. Moreover, most of the 40 validated STs were identified in animals, and to our knowledge, only 14 of them (ST1-ST10, ST12, ST14, ST16, and ST23) have been reported in humans with highly variable frequency [6,23,24,25,26,27,28]. In the human population, more than 90% of the subtyped isolates belong to ST1 to ST4, most likely related to widespread human-to-human transmission [23]. Other STs colonizing humans are less frequent and the presumed result of zoonotic transmission as some of them are, for instance, considered avian (ST6 and ST7) or bovine (ST10 and ST14) STs due to their large predominance in these respective animal groups [2,9,10,14,29,30].

Because of the common asymptomatic carriage of *Blastocystis* sp. in humans, the clinical significance of this enteric protozoan remains a controversial issue. However, various medical case reports [31], together with accumulating in vitro data, have highlighted the pathogenic potential of *Blastocystis* sp. isolates, mainly through the identification of virulence factors. In addition, the demonstration of the damaging effect of this extracellular parasite on the host intestinal epithelium was reported to be ST-dependent [32,33]. Indeed, recent studies have confirmed the hypothesis that *Blastocystis* sp. ST4 is a beneficial commensal by favorably modulating the host gut bacterial community both in vitro and in a murine model [34,35], while ST7 infection is associated with lower bacterial diversity and altered microbial structure in diarrheal patients [36]. Therefore, human infections by *Blastocystis* sp. have been associated with non-specific gastrointestinal disorders, notably including diarrhea, abdominal pain [8], and likely urticaria [37].

Given the potential public health impact of *Blastocystis* sp., numerous epidemiological surveys have been conducted all over the world in the last few years to determine the prevalence and ST distribution of the parasite with the aim of clarifying its circulation. Strikingly, the African continent remains under-investigated, even if a large portion of the African population still lives without access to clean water and sanitary facilities [38]. Therefore, the possible contamination of drinking water with enteric parasites such as *Blastocystis* sp. poses a serious threat. For example, the observed prevalence of *Blastocystis* sp. in the human population can reach an average of 80% in West African countries such as Senegal [5] and Guinea [6]. Concerning North Africa, molecular data related to this geographical area are still insufficient and especially restricted to Egypt, as recently partially reviewed [6]. To our knowledge, nearly twenty studies conducted in this country refer to the subtyping of *Blastocystis* sp. isolates. However, the subtyping was almost exclusively performed after the identification of the parasite in stool samples using conventional methods, including direct light microscopy of smears and/or short-term xenic in vitro stool cultures. Consequently, no accurate human prevalence value based on molecular method has yet been reported in Egypt, which nevertheless ranks as the third most populated African country. In addition, the current number of reported subtyped isolates remains still too limited to define a reliable ST distribution of *Blastocystis* sp. in this country [6]. Therefore, the largest molecular epidemiological survey ever performed in Egypt was conducted on a cohort of more than 800 individuals living in 5 governorates located in the north of the country. Stool samples of participants were screened for the presence of *Blastocystis* sp. via a real-time polymerase chain reaction (qPCR) assay to determine its prevalence. Subsequently, the direct sequencing of the positive PCR products for subtyping of the isolates allowed us to determine the distribution of STs in this Egyptian cohort and then propose transmission routes of the parasite within this population.

## 2. Results and Discussion

To our knowledge, this study represents one of the most comprehensive assessments of the molecular epidemiology of *Blastocystis* sp. in North Africa and more specifically in the Egyptian population. In this context, 825 stool samples of Egyptian individuals with an age range between 1 month and 70 years (mean age of 24.7 ± 17.01 years) and residing in five governorates of Northern Egypt (Alexandria, Beheira, Cairo, Dakahlia, and Ismailia) were examined for the presence of *Blastocystis* sp. The overall prevalence of this parasite determined via the qPCR assay was 72.4% (597/825) (Table 1).

As expected, this prevalence value of 72.4% was higher than those reported in other previous surveys conducted in Egypt, which ranged from 15.4% to 63.0% [39,40,41,42,43,44,45,46,47,48,49,50,51]. Indeed, in these latter studies, the parasite frequency was determined via direct microscopy of fecal smears or after in vitro cultivation of stool samples, which are conventional detection methods known to significantly underestimate the true incidence of the parasite compared to molecular assays [52,53]. Recently, the prevalence of 64.0% and 37.3% have been recorded by PCR for two limited cohorts of Egyptians [54,55], but either almost all or all PCR products obtained in these surveys were not sequenced to confirm the presence of the parasite. Overall, the prevalence observed in our Egyptian cohort was similar to that determined, for instance, in large groups of individuals in West Africa, like in Senegal (80.4%) [5] or Guinea (78.0%) [6]. By further analyzing these epidemiological data from the Egyptian governorate (Table 1), the prevalence of *Blastocystis* sp. was shown to be highly variable, ranging from 41.3% to 100%. Consequently, the parasite was significantly more prevalent in the Alexandria governorate (prevalence of 100%; OR: 77.283, CI: 77.282–77.284, *p* = 0.00050) and less frequent in the Ismailia governorate (prevalence of 41.3%; OR: 0.188, CI: 0.128–0.274, *p* < 0.0001) than in the other Egyptian governorates. This variation in parasite carriage among different communities within the same country has also been observed in numerous surveys, such as in Asian and African countries [1,5,6], and is likely related to a variety of factors, including geographical location, socioeconomic development, subpopulation composition, health status, climate, sanitary conditions, and exposure to environmental and animal sources of contamination. To date, the present survey is the third to describe an impressive 100% prevalence of *Blastocystis* sp. in a human cohort after those reported in two groups of school children in Senegal living in the village of Ndiawdoune or in the Podor disctrict [5,56]. Together with the average prevalence exceeding 70% reported herein, these epidemiological data highlighted the widespread burden and active circulation of *Blastocystis* sp. in Egypt but also more globally in the African population, mainly in link with the fecal peril.

In the present study, stool samples were collected from 404 males (49.0%) and 421 females (51.0%) for a sex ratio (F/M) close to 1. Regarding gender, the difference in prevalence observed between males and females colonized by *Blastocystis* sp. (71.8% versus 72.9%) was not statistically supported (Fisher exact test, *p* = 0.755). Neither age (Fisher exact test, *p* = 0.277) nor contact with animals (Fisher exact test, *p* = 0.0622) were also significantly associated with *Blastocystis* sp. infection. However, individuals in contact with animals were noticeably more colonized (145/186, 78.0%) than those reporting no animal contact (452/639, 70.7%), which could possibly be explained by the well-known zoonotic potential of the parasite [13,14,15].

Within the Egyptian cohort, 203 participants were considered symptomatic by reporting either diarrhea (n = 99), abdominal pain (n = 164), or both digestive symptoms simultaneously (n = 57). Although there was a slight trend towards a higher proportion of individuals colonized with *Blastocystis* sp. in the symptomatic group compared to the asymptomatic category (76.4% versus 71.1%), this association was not statistically significant (Fisher’s exact test, *p* = 0.149). Similarly, the prevalence of *Blastocystis* sp. was moderately higher in participants reporting either diarrhea or abdominal pain with respect to individuals without each of these two symptoms (77.8% versus 71.6% and 73.8% versus 72.0%, respectively) but not significant (Fisher’s exact tests, *p* = 0.231 and *p* = 0.697, respectively). In parallel, stool samples obtained from participants followed in clinic laboratories were significantly more colonized by *Blastocystis* sp. than those collected from participants’ households (75.4% versus 69.2%; OR: 1.368, CI: 1.008–1.860, *p* = 0.045). As the participants referred to the clinic laboratories suffered from various pathologies, potentially including digestive disorders, this could explain the difference in prevalence observed between these two latter groups of individuals.

The qPCR products of the 597 positive samples were then purified and directly sequenced, followed by the analysis of the corresponding sequence chromatograms. Strikingly, a total of 235 sequence patterns (39.4%) were characteristic of mixed infections with double traces, suggesting the presence of at least two *Blastocystis* sp. STs in the same sample (Table 1). A significant proportion of mixed infections have also been recently reported in other African countries, such as Senegal (23.0%) [5] and Guinea (25.4%) [6], undoubtedly associated with the presence of multiple sources of contamination, whether human, animal, or environmental, in geographical areas strongly affected by the parasite. The remaining 362 qPCR products corresponded to single *Blastocystis* sp. infections by one ST and exhibited 99.3% to 100% sequence identity with the available homologous sequences of known STs, allowing their subtyping. The three main STs were represented by ST3 (175/362, 48.3%), ST1 (143/362, 39.5%), and ST2 (39/362, 10.8%) (Table 1). Other than these three STs, a single case of ST10 infection (0.3%) was identified, as well as four individuals colonized by ST14 (1.1%). Considering only the three major STs highlighted within the Egyptian community (ST1 to ST3), no significant association was identified between the ST distribution and age, sex, digestive symptoms, and health status of the participants or contact with animals. In contrast, substantial variations in this ST distribution were reported according to the geographical area studied, with ST3 largely predominant in the governorates of Alexandria, Beheira, and Cairo and ST1 overabundant in the two remaining governorates of Dakahlia and Ismailia. Consequently, the risk of ST1 infection was significantly higher in the Dakhalia governorate (OR: 2.273, CI: 1.553–3.337, *p* < 0.0001) and lower in the Cairo governorate (OR: 0.338, CI: 0.138–0.710, *p* = 0.008) than in the three others governorates. In case of ST3, it was more predominant in Alexandria (OR: 1.819, CI: 1.128–2.907, *p* = 0.013) and Beheira (OR: 3.132, CI: 2.130–4.616, *p* < 0.0001) governorates and less frequently reported in Dakahlia (OR: 0.301, CI: 0.197–0.452, *p* < 0.0001) and Ismailia (OR: 0.403, CI: 0.182–0.801, *p* = 0.0152) governorates. Regarding ST2, no significant association was identified with any of the Egyptian governorates. Globally, the ST distribution highlighted in the present Egyptian cohort was comparable to that detected in a vast majority of countries around the world, with a predominance of ST3 followed by ST1 or ST2, depending on the country [5,23]. This distribution was confirmed by analyzing all the subtyping data available in the literature for Egypt (Table 2), showing that ST3 accounts for about 60% of the 1107 isolates subtyped so far.

Moreover, in other Northern African countries, ST3 also predominated in Tunisia [62] and Algeria [63], whereas ST1 was more common in Libya [23,65], even if these trends need to be confirmed with larger cohorts. It is worth mentioning that ST1 to ST3 represented over 95% of all subtyped isolates in Africa [5], which is almost also the case in Egypt (Table 2).

**Figure 1:**
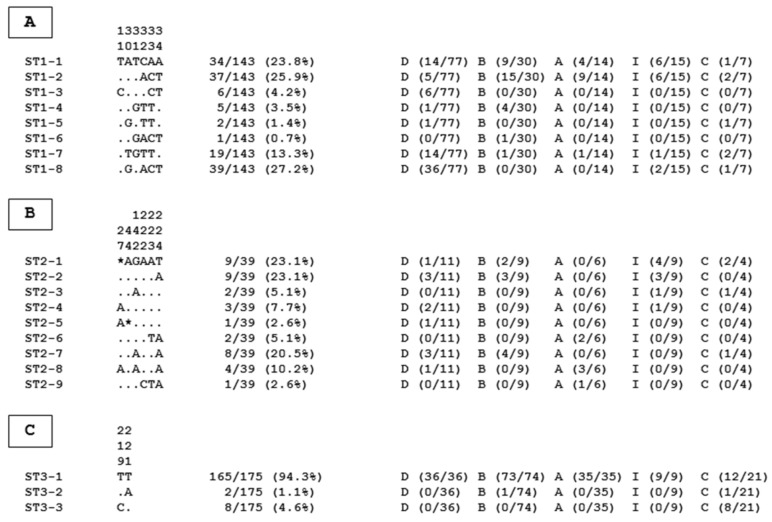
Variable positions identified between partial SSU rRNA gene sequences obtained in the present study from isolates of ST1 (**A**), ST2 (**B**), and ST3 (**C**). The positions of variable positions are indicated above the alignment (vertical numbering) with respect to the arbitrarily selected reference sequences called genotypes ST1-1, ST2-1, and ST3-1. Nucleotides identical to those of these reference sequences are represented by dashes, and gaps are represented by asterisks. All the genotypes identified for each ST are indicated on the left of the alignments. The total number and percentage of isolates identified in our study for each genotype, followed by their repartition per governorate (number of isolates in parentheses), are reported on the right of the alignments. Abbreviations: D, Dakahlia; B, Beheira; A, Alexandria; I, Ismailia; C, Cairo.

To complement these epidemiological data and in order to identify the so-called genotypes present in the Egyptian cohort and assess the intra-ST diversity, the sequence polymorphism of isolates belonging to the major ST1 to ST3 was analyzed by aligning the partial SSU rRNA gene sequences belonging to the same ST with each other (Figure 1).

Through the identification of variable positions in these alignments, eight (ST1-1 to ST1-8), nine (ST2-1 to ST2-9), and three (ST3-1 to ST3-3) genotypes were identified for ST1, ST2, and ST3, respectively. By calculating the average ratio of the number of isolates per genotype, which is inversely proportional to intra-ST variability, ST3 was by far the ST exhibiting the lowest intra-ST diversity, with an average ratio of 58.3 isolates per genotype (175/3), followed by ST1 (average ratio of 17.9 isolates/genotype, 143/8) then ST2 (average ratio of 4.3 isolates/genotype, 9/39). Strikingly, based on the same domain of the SSU rRNA gene, the lowest diversity of ST3 compared to ST1 and ST2 has also been recently highlighted in various human cohorts from Senegal [5], Guinea [6], Syrian refugees in Lebanon [25], and even Vietnam [28].

Together, the subtyping and genotyping data obtained herein allowed us to propose the most convincing hypotheses regarding modes of transmission of *Blastocystis* sp. in the Egyptian cohort. Regarding ST3, its predominance, together with the very restricted number of ST3 genotypes identified, highlighted a wide human-to-human transmission within the cohort, even if it is well known that it can potentially colonize some animal groups [1,2,9,10], even in Egypt [30,41]. Incidentally, the ST3-1 genotype, which represented nearly 95% of ST3 isolates, was predominant in the five governorates studied, reinforcing this statement and suggesting that the same isolate was circulating very actively in the human population throughout Northern Egypt.

The circulation picture was slightly more challenging for ST1 and ST2. Indeed, the high number of genotypes identified for these 2 STs (17 in total) suggested multiple reservoirs of contamination for ST1 and ST2. Although anthroponotic transmission was likely predominant for these two STs [23], zoonotic infection through direct contact with animals could not be excluded since these STs have also been identified in various animals [1,2,9,10]. Water sources contaminated by animal or human feces could also represent additional reservoirs of ST1 and ST2. In this respect, ST1 and ST2 were frequently reported in water sources worldwide [2], and ST2 was, to date, the only ST identified in Egyptian water samples in Dakahlia governorate (Northern Egypt) [66]. Additionally, recreational water tested in Alexandria governorate for the presence of *Blastocystis* sp. revealed a high parasitic contamination [67]. Future studies should be conducted to detect the presence of the parasite in environmental samples collected from the different governorates.

As stated above, the remaining isolates subtyped in the present study belonged to ST10 and ST14, with a cumulative prevalence of 1.4% (5/362) among single *Blastocystis* sp. infections. Our study was the first report of these two STs in the human population in North Africa (Table 2), thus expanding the catalog of STs circulating in this part of the world. Until very recently, these two STs had never been identified in the human population but were predominant in cattle worldwide [1,2,9,10], including Northern Egypt [30], and considered to be likely adapted to bovid [9,29]. Recent epidemiological surveys have reported these two STs in humans in various countries, including Senegal [5], Guinea [6], and Vietnam [28], with variable frequencies. Therefore, the colonization of human cohorts by ST10 and/or ST14 could thus be naturally explained by zoonotic transmission through direct contact with bovid or the consumption of water or food contaminated by animal feces. To further clarify the transmission dynamics of *Blastocystis* sp. in Northern Egypt, epidemiological investigations have to be conducted in the short term through a “One Health” approach that would allow screening of human, animal, and environmental samples collected in a restricted geographical area, as already successfully performed, for instance, in a recent survey in Thailand [27].

## 3. Materials and Methods

### 3.1. Study Area and Sample Collection

**Figure 2:**
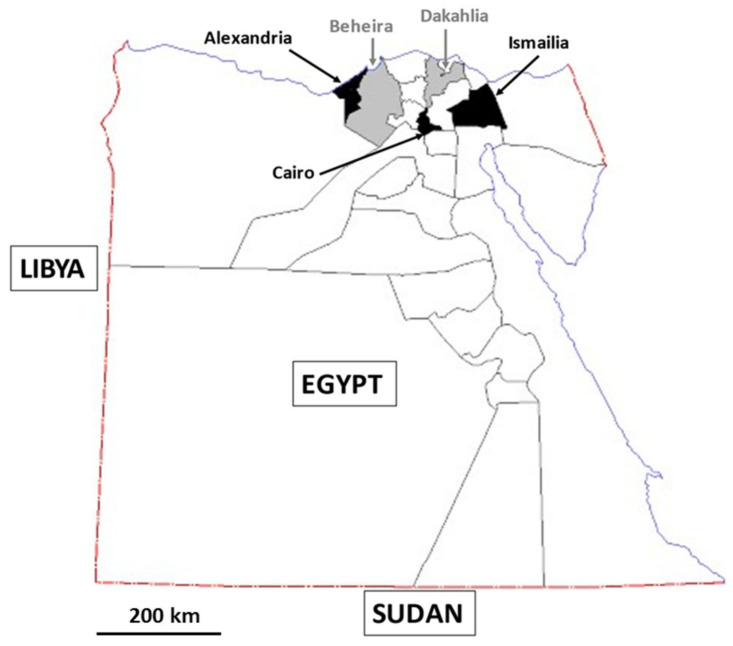
Detailed location of the 5 governorates in Northern Egypt screened for the presence of *Blastocystis* sp.

With a population of over 100 million inhabitants, Egypt is currently the 14th most populated country in the world and the third-most populated in Africa, behind Nigeria and Ethiopia. This North African country (Figure 2) is bordered by the Red Sea to the East, the Mediterranean Sea to the north, Sudan to the south, and Libya to the west. Egypt lies primarily between latitudes 22° and 32° N and longitudes 25° and 35° E and is divided into 27 governorates. The most populous governorate in Egypt is Cairo, which, along with Alexandria, is known as an urban governorate, while Beheira, Dakahlia, and Ismailia are considered rural governorates. Cairo is the capital and largest city of Egypt, while Alexandria, the second-largest city, is an important industrial center.

This large-scale epidemiological survey was conducted over a 6-month period from January 2022 to June 2022. A total of 825 fresh stool samples were collected from participants (1 sample per individual) living in 5 governorates of Northern Egypt, including Alexandria (n = 86), Beheira (n = 202), Cairo (n = 93), Dakahlia (n = 301), and Ismailia (n = 143) (Figure 1). The difference in the number of samples from one governorate to another was related to the varying proportion of volunteers. These governorates were selected on the one hand because of their proximity to Mansoura University (Dakahlia governorate) to facilitate sample collection and storage, and on the other hand, to globally cover in our survey governorates considered as rural or urban as described above. Prior to enrolment, each adult or each parent or guardian of a minor who wished to voluntarily participate in the present study provided signed informed consent after having been fully informed of the aims of the survey. Once enrolled, each participant completed a carefully crafted questionnaire to collect epidemiological data, including gender, age, residence place, source of stool sample (home or clinic laboratory), contact with animals, and presence of gastrointestinal symptoms. The individual was classified as symptomatic if diarrhea and/or abdominal pain were present. Identities of the participants and collected data have been strictly anonymized.

The samples were obtained from clinic laboratories (422 specimens) or directly from participants’ households (403 samples). Briefly, stools from individuals followed up for different pathologies or routine check-ups, with/without gastrointestinal symptoms, were analyzed in clinic laboratories for the detection of potential parasites. After examination, the remaining stool samples were provided from these participants. Specimens were also collected at the participants’ homes using an applicator stick and a clean plastic container. To encourage the recruitment of these volunteers, the family members or neighbors of the participants followed in the clinic laboratories were contacted and informed of every step of the procedure, including the required volume of stool samples as well as the collection and transport of specimens. After shaking around 2 g of each stool specimen in 2 mL of 2.5% potassium dichromate (Sigma Life Sciences, Saint-Louis, MO, USA) for conservation at Mansoura University, all the samples were homogenized then refrigerated at 4 °C until being shipped to the Institut Pasteur of Lille (France) for subsequent analysis.

### 3.2. DNA Extraction, PCR Assay, and Molecular Subtyping of Isolates

Stool diluted in potassium dichromate was stirred and then washed three times with distilled water via centrifugation for 10 min at 3000× *g*. The supernatant was discarded, and the remaining stool pellet was diluted with 1 mL of sterile water. The genomic DNA was extracted using the NucleoSpin 96 Soil kit (Macherey-Nagel GmbH & Co KG, Düren, Germany) from 500 µL of the diluted pellet according to the manufacturer’s recommended procedures. DNA extraction negative control was also included. DNA was then eluted with 100 µL of elution buffer provided in the DNA extraction kit and stored at −20 °C until PCR analysis. *Blastocystis* sp. was detected in every specimen via qPCR assay targeting a domain of the SSU rRNA gene using 2 μL of the extracted DNA and *Blastocystis*-specific primer pair BL18SPPF1 (5′-AGTAGTCATACGCTCGTCTCAAA-3′)/BL18SR2PP (5′-TCTTCGTTACCCGTTACTGC-3′), as described previously [52]. Each sample was analyzed in duplicate, using the DNA obtained from an axenic culture of *Blastocystis* sp. ST8 as the positive control and reagent-grade water and DNA extraction control as the negative controls for the qPCR assay. The amplified fragment of the SSU rRNA gene with a length of approximately 300 bp is sufficiently discriminating in terms of sequence information to subtype the *Blastocystis* sp. isolates [5,6,25,52]. All the positive qPCR products were purified and then directly sequenced in both directions using Genoscreen (Lille, France; SANGER technology platform, 3730XL DNA Analyzer). In the case of a significant proportion of specimens, the sequence chromatograms showed double traces reflecting mixed infections (presence of at least two different STs of *Blastocystis* sp. in the same sample). For these positive samples, the STs colonizing the corresponding participants were not determined. The sequences obtained in this study from individuals with single *Blastocystis* sp. infections have been deposited in GenBank under accession numbers OQ469844-OQ470205. For subtyping of isolates, these sequences were compared with all homologous *Blastocystis* sp. sequences of known mammalian and avian STs available from the National Center for Biotechnology Information (NCBI) using the nucleotide Basic Local Alignment Search Tool (BLASTn) program. The sequences of *Blastocystis* sp. isolates belonging to the same ST were subsequently aligned against each other using the BioEdit v. 7.0.1 package (date of release: 6 October 2019). (http://www.mbio.ncsu.edu/BioEdit/bioedit.html) to determine the genetic relatedness and intra-ST diversity and recognize so-called genotypes proposed in previous surveys [5,6,25,28], which are representative of genetically distinct strains within the same ST.

### 3.3. Statistical Analysis

Fisher’s exact test was used to study the relationship between the various category variables (gender, age, and place of residence of the participants; source of sample collection; animal contact; presence of gastrointestinal symptoms; and health status). The prevalence of *Blastocystis* sp. and STs were chosen as the main outcomes, and logistic regression models were used to generate odds ratios (OR) and the 95% confidence interval (CI). In order to deal with the zero-frequency statistical issue related to the 100% prevalence of the parasite reported in the Alexandria governorate, a Laplace additive smoothing technique was first applied to the observation counts. A *p*-value of less than 0.05 was set as the limit for significance with a 95% confidence interval. The analyses were conducted using the package statistics and odds ratio 2.0.1 from R statistical computing software (version 4.1.1; release date: 10 August 2021; R Development Core Team, http://www.R-project.org, accessed on 10 June 2022).

## 4. Conclusions

To the best of our knowledge, this study represents the largest molecular epidemiological survey ever conducted in North Africa and provides insight into the impact, circulation, and transmission of *Blastocystis* sp. in this under-investigated region of the world. The prevalence observed in Egypt exceeds 70%, highlighting a very active circulation of this parasite in this country, mainly due to widespread human-to-human transmission but also to various environmental and animal reservoirs, which need to be identified in further studies to understand their role in the spread of the parasite. Such a high prevalence of *Blastocystis* sp. in the Egyptian population emphasizes the importance of routine screening and implementation of preventive public health and hygiene programs for the population. Indeed, besides its potential virulence and because of its high prevalence worldwide and fecal-oral transmission, *Blastocystis* sp. can be considered a good sentinel of environmental health quality and water contamination. As such, its absence will also highlight the absence of other fecal–oral transmitted intestinal parasites known to be pathogens and present in the same environment. These measures may thus assist health authorities in reducing the prevalence and the risk of intestinal parasites in combination with improved sanitation to minimize possible contact with contaminated water resources.

## Figures and Tables

**Table 1:** Prevalence and ST distribution of *Blastocystis* sp. reported in the present study in five governorates of Northern Egypt.

				*Blastocystis* sp. STs					
Governorate	Samples (n)	Positive Samples (n)	Prevalence (%)	ST1	ST2	ST3	ST10	ST14	MI a
Alexandria	86	86	100	14	6	35	0	0	31
Beheira	202	154	76.2	30	9	74	0	1	40
Cairo	93	67	72.0	7	4	21	0	1	34
Dakahlia	301	231	76.7	77	11	36	1	2	104
Ismailia	143	59	41.3	15	9	9	0	0	26
**Total**	**825**	**597**	**72.4**	**143**	**39**	**175**	**1**	**4**	**235**

^a^ MI, mixed infections.

**Table 2:** Prevalence and distribution of *Blastocystis* sp. STs in North African countries.

Countries	Prevalence	Identification Method	Number of Subtyped Isolates	Subtyping Method	*Blastocystis* sp. STs									MI ^a^	Reference
					ST1	ST2	ST3	ST4	ST5	ST6	ST7	ST10	ST14		
Egypt	34.5%	DLM ^b^	36	PCR-STS ^c^	6	0	30	0	0	0	0	0	0	0	[39]
Egypt	19.1%	XIVC ^d^	22	Sequencing	4	0	18	0	0	0	0	0	0	0	[40]
Egypt	35.7%	XIVC ^d^	53	PCR-STS ^c^	16	4	30	3	0	0	0	0	0	0	[41]
Egypt	63.0%	DLM ^b^	20	RFLP ^e^	4	2	11	3	0	0	0	0	0	0	[42]
Egypt	39.0%	DLM ^b^ + XIVC ^d^	6	Sequencing	2	3	1	0	0	0	0	0	0	0	[43]
Egypt	23.1%	XIVC ^d^	44	PCR-STS ^c^	8	0	24	0	0	8	4	0	0	0	[44]
Egypt	15.4%	DLM ^b^	11	Sequencing	3	3	5	0	0	0	0	0	0	0	[45]
Egypt	33.0%	DLM ^b^	100	RFLP ^e^	0	0	84	16	0	0	0	0	0	0	[46]
Egypt	18.2%	XIVC ^d^	2	PCR-STS ^c^	1	0	1	0	0	0	0	0	0	0	[47]
Egypt	54.2%	DLM ^b^	51	PCR-STS ^c^	9	2	40	0	0	0	0	0	0	0	[48]
Egypt	52.0%	XIVC ^d^	20	Sequencing	5	4	8	0	0	0	3	0	0	0	[49]
Egypt	39.4%	DLM ^b^	63	PCR-STS ^c^	10	0	29	15	0	0	0	0	0	9	[50]
Egypt	60.4%	PCR ^f^	10	Sequencing	3	3	4	0	0	0	0	0	0	0	[54]
Egypt	NA ^g^	DLM ^b^	21	Sequencing	4	4	13	0	0	0	0	0	0	0	[57]
Egypt	NA ^g^	XIVC ^d^	110	PCR-STS ^c^	15	0	49	0	0	33	13	0	0	0	[58]
Egypt	NA ^g^	XIVC ^d^	33	RFLP ^e^	0	0	33	0	0	0	0	0	0	0	[59]
Egypt	NA ^g^	XIVC ^d^	60	PCR-STS ^c^	23	2	35	0	0	0	0	0	0	0	[60]
Egypt	NA ^g^	XIVC ^d^	102	RFLP ^e^	20	15	57	10	0	0	0	0	0	0	[61]
Egypt	72.4%	qPCR	597	Sequencing	143	39	175	0	0	0	0	1	4	235	**Present study**
**Total**			**1361**		**276**	**81**	**647**	**47**	**0**	**41**	**20**	**1**	**4**	**244**	
Tunisia	NA ^g^	DLM ^b^	61	Sequencing	18	10	31	1	0	0	1	0	0	0	[62]
Algeria	7.4%	DLM ^b^	30	Sequencing	10	4	15	1	0	0	0	0	0	0	[63]
Algeria	11.6%	DLM ^b^	3	Sequencing	0	2	1	0	0	0	0	0	0	0	[64]
Libya	28.0%	XIVC ^d^	38	Sequencing	19	3	15	0	0	0	1	0	0	0	[23]
Libya	22.1%	XIVC ^d^	48	Sequencing	26	13	9	0	0	0	0	0	0	0	[65]
**Grand total**			**1541**		**349**	**113**	**718**	**49**	**0**	**41**	**22**	**1**	**4**	**244**	

^a^ Mixed infections with undetermined STs. ^b^ DLM, direct-light microscopy of smears. ^c^ STS, subtype-specific sequence-tagged site. ^d^ XIVC, Xenic in vitro stool culture. ^e^ RFLP, Restriction Fragment Length Polymorphism. ^f^ PCR assay not followed by sequencing of all PCR products to confirm the presence of the parasite. ^g^ NA, not applicable.

## Data Availability

All relevant data are within the paper.
